# The role of diet in moderating the relationship between symptoms of depression and brain amyloid load

**DOI:** 10.1002/alz.70560

**Published:** 2025-08-07

**Authors:** Hilal Salim Said Al Shamsi, Samantha L. Gardener, Stephanie R. Rainey‐Smith, Hamid R. Sohrabi, Kevin Taddei, Colin L. Masters, Vincent Doré, Christopher Rowe, W. M. A. D. Binosha Fernando, Ralph N. Martins, for the AIBL Research Group

**Affiliations:** ^1^ Centre of Excellence for Alzheimer's Disease Research and Care, School of Medical and Health Sciences Edith Cowan University Bentley Western Australia Australia; ^2^ Alzheimer's Research Australia Ralph and Patricia Sarich Neuroscience Research Institute Nedlands Western Australia Australia; ^3^ Lifestyle Approaches Towards Cognitive Health Research Group Murdoch University Murdoch Western Australia Australia; ^4^ Centre for Healthy Ageing Health Futures Institute Murdoch University Murdoch Western Australia Australia; ^5^ School of Psychological Science University of Western Australia Perth Western Australia Australia; ^6^ Department of Biomedical Sciences Macquarie University Sydney New South Wales Australia; ^7^ Department of Health and Biosecurity Flagship The Australian eHealth Research Centre, Commonwealth Scientific and Industrial Research Organisation Parkville Victoria Australia; ^8^ Department of Molecular Imaging Austin Health Heidelberg Victoria Australia; ^9^ The Florey Institute of Neuroscience and Mental Health The University of Melbourne Parkville Victoria Australia

**Keywords:** Alzheimer's disease, amyloid beta, anxiety, depression, dietary patterns

## Abstract

**BACKGROUND:**

Healthy lifestyle factors, including diet, may affect brain amyloid beta (Aβ) load. This study examines dietary patterns as moderators of the relationships among symptoms of depression, anxiety, and brain Aβ load.

**METHOD:**

A cross‐sectional study of cognitively unimpaired older adults (*n* = 524) from the Australian Imaging, Biomarkers, and Lifestyle study assessed dietary patterns, depressive and anxiety symptoms, and brain Aβ load. Moderation and simple slope analyses were conducted.

**RESULTS:**

The Dietary Approaches to Stop Hypertension (DASH) diet moderated the relationship between depressive and anxiety symptoms and brain Aβ load. Higher symptoms were associated with greater Aβ load in individuals with lower DASH adherence. This effect was also observed for anxiety symptoms in apolipoprotein E ε4 carriers. The Mediterranean and Western diets did not moderate these relationships.

**CONCLUSION:**

The DASH diet adherence may mitigate the impact of depressive and anxiety symptoms on brain Aβ load, supporting genotype‐specific dietary interventions in mental and brain health.

**Highlights:**

- The Dietary Approaches to Stop Hypertension (DASH) diet moderates the links among depression, anxiety, and brain amyloid load.
- Higher symptoms were linked to greater amyloid load in those with low DASH adherence.
- This effect was observed for anxiety symptoms in apolipoprotein E ε4 allele carriers.
- Mediterranean and Western diets did not moderate these relationships.
- Findings support genotype‐specific dietary interventions for brain and mental health.

## 1 BACKGROUND

Depression and anxiety, prevalent mental health conditions, are both potential risk factors and symptoms of Alzheimer's disease (AD), possibly accelerating disease progression.^1^, ^2^, ^3^ Although the exact mechanisms remain unclear, several have been proposed.^4^, ^5^, ^6^ AD is characterized by amyloid beta (Aβ) plaques and tau tangles, which damage neurons in key areas of the brain that regulate mood, such as the amygdala and prefrontal cortex, potentially triggering anxiety and depressive symptoms.^7^ AD also disrupts the stress response system, further contributing to these symptoms.^7^ Cognitive decline and loss of independence often lead to sadness and hopelessness,^8^ exacerbated by isolation and stigma.^7^, ^9^, ^10^ Approximately 50% of AD patients experience depression,^11^, ^12^, ^13^ which has been linked to future cognitive decline and disease progression.^14^

Evidence suggests AD hallmarks—Aβ plaques and tau tangles—may be linked to depression and anxiety symptoms, increasing AD risk.^6^ Wu et al.^15^ found participants with a history of major depressive disorder (MDD) had higher Aβ burden in the precuneus (*p* = 0.045) and parietal region (*p* = 0.038) than those without depression.^15^ A further study found significantly higher combined tau and Aβ levels in MDD patients (*p* = 0.01) and specifically in the posterior cingulate (*p* = 0.04) and lateral temporal regions (*p* = 0.01).^16^ Lavretsky et al.^17^ assessed 43 dementia‐free participants (23 had mild cognitive impairment [MCI]), examining depression, anxiety symptoms, and brain Aβ/tau quantification. In the MCI group, depressive symptoms were associated with tau and Aβ binding in the lateral temporal region, while anxiety symptoms correlated with the posterior cingulate. In non‐MCI participants, depression and anxiety symptoms were linked to tau/Aβ binding in the medial temporal and frontal regions.^17^ These findings suggest even mild symptoms are linked to Aβ/tau deposition, with variations depending on the level of cognitive impairment. Conversely, Madsen et al.^18^ found no association between depressive episodes and Aβ burden; however, their analysis focused on individuals recovered from depression at the time of Aβ imaging.

Research has increasingly shown that lifestyle factors, including diet, have a significant impact on brain function and mental well‐being.^19^ The Mediterranean diet (MeDi) and the Dietary Approaches to Stop Hypertension (DASH) diet are linked to reducing the risk and progression of depression and AD.^20^, ^21^, ^22^ Rich in fruits, vegetables, fish, and nuts, they provide antioxidants, omega‐3 fatty acids, and anti‐inflammatory foods that combat oxidative stress and inflammation, key factors in depression and AD.^23^, ^24^, ^25^ In contrast, a Western diet, high in refined grains and processed foods, is associated with higher depression rates^26^, ^27^ and an increased AD risk,^28^ highlighting the role of nutrition in mental and cognitive health.

The role of diet in the relationships among symptoms of depression, anxiety, and AD pathology remains unclear. While certain dietary patterns may influence mental health, the underlying mechanisms need further exploration. Researchers are investigating how specific nutrients and food groups might interact with brain health and mood disorders, with genetics and lifestyle factors adding complexity. A comprehensive understanding of dietary effects on disease progression is crucial for developing effective preventive interventions. To address this gap, we examined the moderating effects of dietary patterns on the relationship between symptoms of depression and anxiety, and brain Aβ load. We hypothesized that greater adherence to healthier diets and lower adherence to unhealthy diets would mitigate the negative effects of depression and anxiety on brain Aβ load. We also explored whether apolipoprotein E (*APOE*) ɛ4 allele carriers exhibit different moderating effects of diets on these relationships.

## 2 METHODS

### 2.1 Participants

This study was conducted on cross‐sectional data from 524 cognitively unimpaired participants from the Australian Imaging, Biomarker & Lifestyle (AIBL) study of aging^29^, ^30^ who completed the Cancer Council Victoria Food Frequency Questionnaire (CCVFFQ) and had a positron emission tomography (PET) scan at their baseline assessment. All AIBL participants were aged ≥ 60; exclusion criteria included a history of bipolar disorder, schizophrenia, non‐AD dementia, recent cancer (except basal cell skin carcinoma), Parkinson's disease, insulin‐dependent diabetes, symptomatic stroke, uncontrolled diabetes, and excessive alcohol consumption (more than two standard drinks per day for women or four for men). AIBL exclusion criteria also include those exhibiting significant current depression (Geriatric Depression Scale [GDS] score > 5). Further details regarding recruitment, assessment, inclusion, and exclusion criteria have been described in detail elsewhere.^29^, ^30^

The AIBL Study received ethical approval from the institutional ethics committees of Austin Health, St. Vincent's Health, Hollywood Private Hospital (now Ramsay Health Care), and Edith Cowan University.^29^ Written informed consent was obtained from each participant before undergoing any study‐related procedures.

### 2.2 Dietary assessment

The CCVFFQ provides a comprehensive and efficient means of assessing the dietary habits of study participants.^31^ This questionnaire includes 74 dietary items, allowing detailed information on food and nutrient intake over the past 12 months to be obtained. The CCVFFQ captures data on the daily consumption of various food items in grams per day. The food composition data used to calculate daily nutrient intake originates from the Nutrient Data Table for Use in Australia 1995 (NUTTAB95). This database provides detailed nutritional information on a wide range of foods consumed in Australia and was published by the Australian government's Department of Health in 1995.^32^

### 2.3 MeDi score

The MeDi score is determined using a standardized method.^33^ Individuals are assigned a score of 1 for each beneficial component (fruits, vegetables, fish, cereals, and legumes) if their calorie‐adjusted consumption meets or exceeds cohort sex‐specific medians. Conversely, a score of 1 is allocated for detrimental components (meat and dairy products) if their calorie‐adjusted consumption falls below cohort sex‐specific medians. Furthermore, a score of 1 is assigned for a monounsaturated fat to saturated fat ratio at or above the median. Those individuals with moderate alcohol consumption (females: > 5– < 25 g/day, males: > 10–< 50 g/day) also receive a score of 1 for this component. The overall MeDi score is calculated as the sum of these individual scores, ranging from 0 to 9.^33^

### 2.4 DASH diet score

The DASH diet emphasizes reduced consumption of total fat, saturated fat, red meat, sugar, and cholesterol, while advocating for improved intake of vegetables, nuts, fruits, fish, whole grains, low‐fat dairy products, and poultry. The DASH diet score is created “a priori*”* with participants categorized into quintiles based on their intake of nine components: vegetables, fruits, nuts and legumes, whole grains, low‐fat dairy products, sodium, red and processed meats, sweetened beverages, and monounsaturated/polyunsaturated fat intake.^34^ As sweetened beverage intake was not a component assessed by the food frequency questionnaire, this was substituted for sugar intake in our DASH diet construction. Food intake (measured in grams) was adjusted for total energy. For beneficial components, individuals receive 5 points for being in the highest intake quintile, with scores decreasing incrementally to 1 point for the lowest intake quintile (quintile 1). Conversely, for detrimental components such as sugar, sodium, and red and processed meats, 5 points are allocated for quintile 1 (lowest intake), decreasing to 1 point for quintile 5 (highest intake). Subsequently, these component scores are aggregated to provide an overall score ranging from 9 to 45,^34^ with higher scores indicating a healthier diet.

**RESEARCH‐IN‐CONTEXT**

1. **Systematic review**: Literature was reviewed using traditional (e.g., PubMed) sources and meeting abstracts and presentations, identifying studies on dietary patterns, particularly the Dietary Approaches to Stop Hypertension (DASH) diet, and their potential role in reducing the accumulation of hallmark amyloid beta (Aβ) plaques. The role of diet in the relationships among symptoms of depression, anxiety, and AD pathology remains unclear.
2. **Interpretation**: The DASH diet moderated the relationship between depressive and anxiety symptoms and brain Aβ load. Higher symptoms were associated with greater Aβ load in individuals with lower DASH adherence. This effect was also observed for anxiety symptoms in apolipoprotein E ε4 carriers. The Mediterranean and Western diets did not moderate these relationships.
3. **Future directions**: Longitudinal and intervention studies are required to establish causality and assess personalized dietary strategies based on genetic risk profiles. Future research should explore whether dietary modifications can mitigate Aβ burden and enhance cognitive resilience in aging populations.

### 2.5 Western diet score

The a posterior Western dietary pattern involved categorizing 101 food and beverage items from the CCVFFQ into 33 predefined food groups to reduce individual food intake variations. Factor analysis (principal components) was applied using the absolute weight in grams per day for each food group. The resulting factors were rotated for clarity, using the varimax procedure to make them uncorrelated. The number of factors to retain was determined based on eigenvalues (> 1.25), scree tests, and interpretability. A cut‐off of 0.30 was used to determine factor loadings included in the pattern. The Western diet pattern extracted from this analysis and its associated factor loadings is presented in Table 1. Foods with higher factor loadings contribute more to the pattern, characterized by high loadings for red and processed meats, poultry, potatoes, refined grains, sweets and snacks, condiments, pizza, and nuts. For each participant, the Western diet score was calculated by summing the observed intakes of the food items included in the pattern, with each item weighted according to its specific factor loading. This resulted in a composite score representing overall adherence to the Western dietary pattern, for which higher scores reflected greater adherence. This method of scoring has been used in previous research.^21^

**TABLE 1:** Western diet pattern factor loadings from factor analysis.

Western diet pattern	
Factor	Factor loadings
Red meats	0.610
Poultry	0.593
Processed meats	0.569
Potatoes	0.556
Sweets	0.554
Refined grains	0.520
Condiments	0.503
Dark yellow vegetables	0.466
Pizza	0.450
Other breakfast cereals	0.446
Chips	0.445
Snacks	0.397
Nuts	0.374
Fish	0.357

### 2.6 Anxiety and depression symptoms assessment

The Hospital Anxiety and Depression Scale (HADS) was used to assess anxiety and depression symptoms among participants. This widely used self‐report questionnaire measures symptoms of anxiety and depression in various clinical and research contexts. Comprising 14 items—7 for anxiety and 7 for depression—it offers valuable insights into an individual's emotional well‐being. Each item is scored from 0 to 3, with 3 representing the highest level of anxiety or depression. An overall subscale score > 8 indicates significant symptoms of depression or anxiety.^35^

### 2.7 Neuroimaging biomarker analysis—Aβ PET

Brain Aβ load is measured in AIBL participants with PET using one of the following Aβ tracers: ^11^C‐Pittsburgh compound B, ^18^F‐NAV4694, ^18^F‐Florbetaben, ^18^F‐Flutemetamol, or ^18^F‐Florbetapir. Methodology for each tracer has been described previously.^36^ Briefly, PET images are analyzed using CapAIBL, and brain Aβ burden is expressed in the Centiloid (CL) scale, to normalize values from different radiotracers, the methodology of which has been shown to be valid and reliable.^36^ The CL scale provides a single continuous scale across the different Aβ imaging tracers, with a value of 0 representing the typical brain Aβ load in young controls, and 100 the typical brain Aβ load seen in mild AD patients.^37^

### 2.8 *APOE* genotyping

DNA was extracted from whole blood samples using QIAamp DNA blood spin column kits (Qiagen) as previously described.^29^, ^30^ Participants’ *APOE* genotypes were determined using Taq Man genotyping assays (Life Technologies) using the Quant‐StudioTM 12k Flex Real‐Time‐PCR system (Applied Biosystems), as previously described.^38^, ^39^ *APOE* carrier status, as included in data analysis, was defined as the presence (one or two copies) or absence (zero copies) of the ε4 allele.

### 2.9 Statistical analysis

Statistical analyses were carried out using R version 4.3.0, with a significance threshold *p* < 0.05.

Body mass index (BMI) was the only variable with missing data. We used multivariate imputation by chained equations to estimate BMIs for 44 participants. The imputed BMI data closely reflected the initial distribution, justifying its inclusion in subsequent analysis.

The optimal model configuration, determined by the Akaike information criterion (AIC) during the model selection process, identified the best‐fitting model, which included the interaction between brain Aβ and diet scores, alongside covariates sex, age, country of birth (Australia vs. other), and *APOE* ε4 allele carriage status. Among the 159 *APOE* ε4 carriers, 16 were homozygous (ε4/ε4), while the remaining 143 were heterozygous (ε3/ε4 or ε2/ε4). Given the small number of ε4 homozygotes, analyses were conducted using a binary ε4 carrier status. BMI, years of education (≤ 12 years vs. > 12 years; as a proxy for socioeconomic status), energy intake, past smoking status, depression medication, and a cardiovascular disease (CVD) score (ranging from 0–4, participants were given a point for current or previous history of hypertension, stroke, heart attack, and angina) were included in the model selection process but found to not be included in the best‐fitting model.

Means, standard deviations, and percentages are provided for the entire cohort demographics and when stratified by *APOE* genotype. Group relationships were examined through independent samples *t* tests for continuous variables and chi‐squared (χ2) tests for categorical variables.

The primary objective of the analysis was to investigate whether dietary patterns moderate the relationships between symptoms of depression or anxiety and brain Aβ. The moderation analyses were conducted separately for each dietary pattern (MeDi, DASH, and Western diet), with each depression and anxiety symptom assessment, as well as brain Aβ load. The false discovery rate (FDR) correction method was implemented to address the issue of multiple comparisons. Furthermore, the moderating effect was explored using a simple slopes analysis, enabling the visualization of the data at the mean and one standard deviation (SD) above and below the mean of the moderator variable, the diet score.

## 3 RESULTS

### 3.1 Demographic statistics

The cohort comprised 524 cognitively unimpaired participants (44% male) with an average age of 71.74 ± 6.03 years. Nearly 30% were *APOE* ε4 allele carriers (the most common genetic risk factor for AD). Non‐carriers of the *APOE* ε4 allele were significantly older than *APOE* ε4 allele carriers. The *APOE* ε4 allele carriers had significantly higher brain Aβ load than non‐carriers, and a higher percentage were born in Australia (Table 2). There were no statistically significant differences between *APOE* ε4 allele carriers and non‐carriers regarding symptoms of depression and anxiety, or dietary pattern scores.

**TABLE 2:** Descriptive statistics for the cognitively unimpaired cohort and stratified by *APOE* ɛ4 carrier status.

	CU (*n* = 524)	*APOE* ɛ4 carrier (*n* = 159)	*APOE* ɛ4 non‐carrier (*n* = 365)	*p* values for *APOE* ɛ4 carriage differences
Age, y	71.74 ± 6.03	70.47 ± 5.95	72.38 ± 5.96	**<0.001**
Country of birth, Australia, n (%)	379 (72.33)	126 (79.25)	253 (69.32)	**0.038**
Sex, male, n (%)	228 (43.51)	74 (46.54)	154 (42.19)	0.374
Brain Aβ, CL	16.18 ± 33.17	32.47 ± 43.14	8.75 ± 24.10	**<0.001**
Depressive symptoms	2.21 ± 1.90	2.09 ± 1.94	2.27 ± 2.02	0.335
Anxiety symptoms	4.11 ± 2.76	4.04 ± 2.80	4.13 ± 2.76	0.725
MeDi	4.40 ± 1.66	4.50 ± 1.60	4.35 ± 1.67	0.332
DASH diet	27.04 ± 4.30	27.50 ± 4.50	26.79 ± 4.16	0.080
Western diet	187.57 ± 101.44	190.00 ± 106.63	187.55 ± 99.04	0.800

*Notes*: Unless otherwise described, data are presented as mean ± standard deviation of the mean. Characteristics compared using an independent samples t‐test for continuous variables and χ2 for categorical variables.

Bold indicates significance (*p* < 0.05).

Abbreviations: Aβ, amyloid beta; *APOE*, apolipoprotein E; CL, Centiloid; CU, cognitively unimpaired; DASH, Dietary Approaches to Stop Hypertension; MeDi, Mediterranean diet; y, years.

### 3.2 Moderation analysis

**FIGURE 1:**
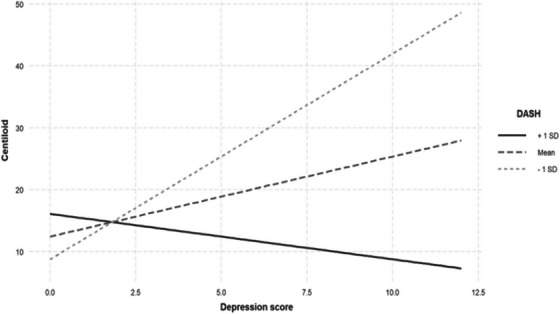
Simple slopes graph of the relationship between symptoms of depression and brain Aβ at levels of DASH diet adherence. Each line represents the association between symptoms of depression and brain Aβ when all datapoints are at either the mean, or 1 SD above, or below, the mean DASH diet adherence. There were no significant associations between symptoms of depression and brain Aβ at 1 SD above the mean and mean DASH diet adherence levels (β = −0.734, SE = 0.960, *p* = 0.445; β = 1.293, SE = 0.681, *p* = 0.058, respectively). At 1 SD below mean DASH diet adherence, greater depressive symptoms were associated with greater brain Aβ (β = 3.321, SE = 0.943, *p* < 0.001). Model includes *APOE* ε4 allele carrier status (±), age, country of birth, and sex as main effects. Aβ, amyloid beta; *APOE*, apolipoprotein E; DASH, Dietary Approaches to Stop Hypertension; SD, standard deviation; SE, standard error.

After adjustment for FDR, a significant interaction between symptoms of depression and the DASH diet score on brain Aβ load was observed (Figure 1, Table 3). Simple slopes analysis revealed that this interaction was significant in participants adhering to the DASH diet score at 1 SD below the mean level with a positive relationship (β = 3.321, standard error [SE] = 0.943, *p* < 0.001), meaning that higher depressive symptoms were associated with higher brain Aβ load (as shown by a higher CL scale) among participants consuming the DASH diet below the mean level. At the mean level and 1 SD above the mean level of the DASH diet, the relationship between depression and brain Aβ load was not significant (β = 1.293, SE = 0.681, *p* = 0.058; β = –0.734, SE = 0.960, *p* = 0.445, respectively).

**TABLE 3:** Moderation analyses of diet scores on the relationship between symptoms of depression and anxiety on brain Aβ in the cohort as a whole and stratified by *APOE* ɛ4 allele carriage.

			HADS‐D x Diet score				HADS‐A x Diet score			
	Diet score	*APOE* ɛ4 allele carriage	β	SE	*p* value	FDR adjusted *p* value	β	SE	*p* value	FDR adjusted *p* value
Brain Aβ, CL	MeDi		0.047	0.435	0.672	0.913	0.299	0.706	0.672	0.672
Brain Aβ, CL	DASH diet		−0.476	0.156	**0.002**	**0.004**	−0.518	0.243	**0.033**	**0.033**
Brain Aβ, CL	Western diet		0.003	0.007	0.678	0.673	−0.005	0.004	0.294	0.588
Brain Aβ, CL	MeDi	+	0.892	1.264	0.483	0.642	−0.358	0.829	0.666	0.666
Brain Aβ, CL	MeDi	–	−0.534	0.389	0.643	0.514	−0.392	0.264	0.138	0.552
Brain Aβ, CL	DASH diet	+	−0.704	0.397	0.078	0.135	−0.719	0.260	**0.006**	**0.024**
Brain Aβ, CL	DASH diet	–	−0.235	0.143	0.101	0.135	−0.123	0.103	0.234	0.234
Brain Aβ, CL	Western diet	+	−0.017	0.015	0.244	0.459	−0.009	0.012	0.459	0.459
Brain Aβ, CL	Western diet	–	0.010	0.007	0.159	0.459	−0.004	0.004	0.373	0.459

*Notes*: Models include age, country of birth, and sex as main effects.

Bold indicates significance (*p* < 0.05).

Abbreviations: Aβ, amyloid beta; APOE, apolipoprotein E; CL, Centiloid; DASH, Dietary Approaches to Stop Hypertension; FDR, false discovery rate; HADS‐A, Hospital Anxiety and Depression Scale for Anxiety; HADS‐D, Hospital Anxiety and Depression Scale for Depression; MeDi, Mediterranean diet; SE, standard error.

**FIGURE 2:**
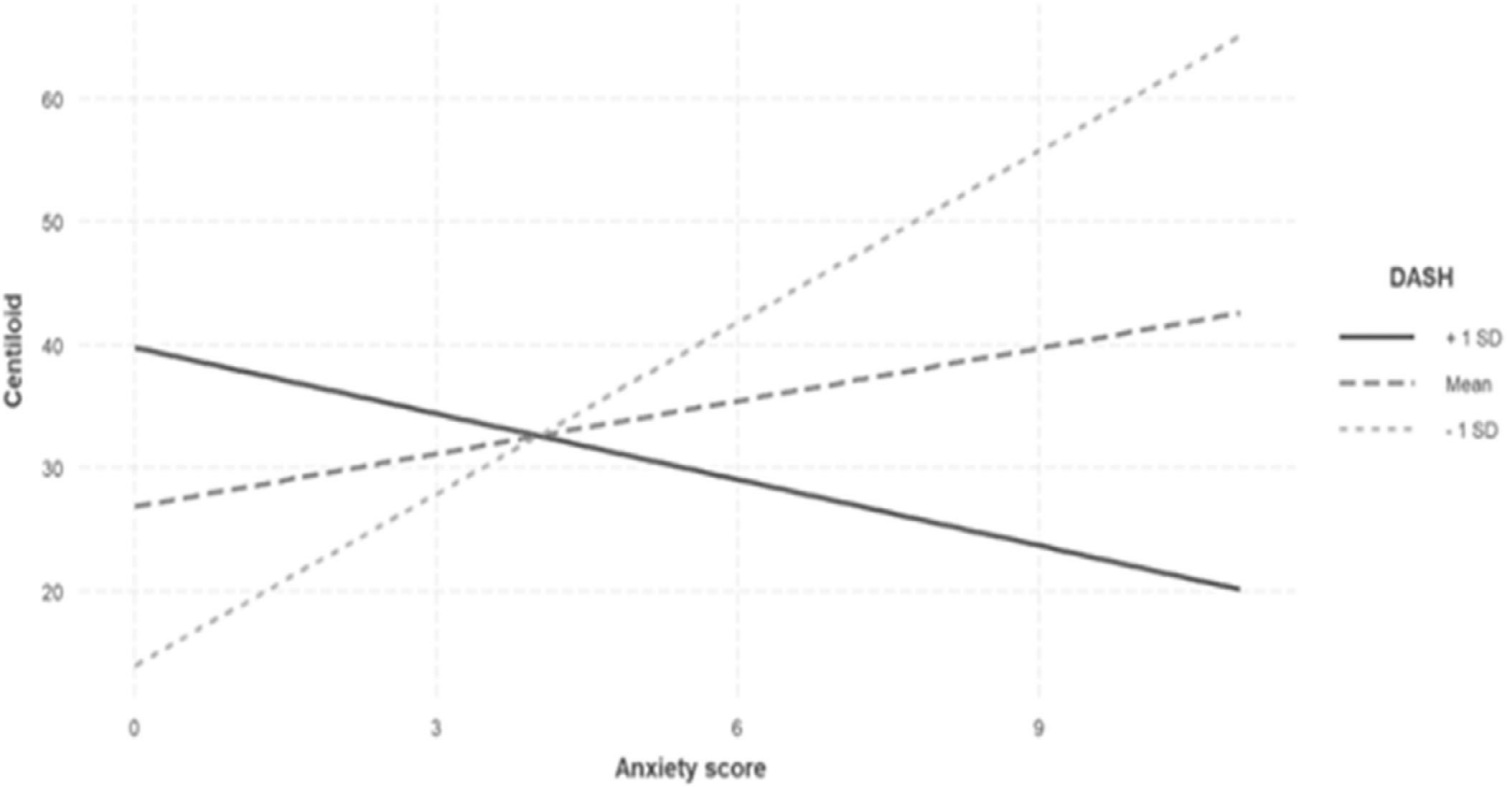
Simple slopes graph of the relationship between symptoms of anxiety and brain Aβ at levels of DASH diet adherence in *APOE* ɛ4 allele carriers. Each line represents the association between symptoms of anxiety and brain Aβ when all data points are at either the mean, or 1 SD above, or below, the mean DASH diet adherence. In *APOE* ɛ4 allele carriers, there were no significant associations between symptoms of anxiety and brain Aβ at 1 SD above mean and mean DASH diet adherence levels (β = −1.787, SE = 1.684, *p* = 0.291; β = 1.543, SE = 1.199, *p* = 0.235, respectively). At 1 SD below mean DASH diet adherence, greater anxiety symptoms were associated with greater brain Aβ (β = 4.465, SE = 1.655, *p* = 0.006). The model includes age, country of birth, and sex as main effects. Aβ, amyloid beta; *APOE*, apolipoprotein E; DASH, Dietary Approaches to Stop Hypertension; SD, standard deviation; SE, standard error.

Similarly, there was a significant interaction between anxiety symptoms and the DASH diet score on brain Aβ load (Table 3). Simple slopes analysis revealed that this interaction was significant again, in participants who adhered to the DASH diet at 1 SD below the mean level with a positive relationship (β = 2.026, SE = 0.679, *p* = 0.003), meaning that higher anxiety symptoms were associated with higher brain Aβ load (as shown by a higher CL scale) among participants consuming the DASH diet below the mean level. At the mean level and 1 SD above the mean level of the DASH diet, the relationship between depression and brain Aβ load was not significant (β = –0.567, SE = 0.490, *p* = 0.247; β = –0.892, SE = 0.665, *p* = 0.180, respectively). However, upon stratification by *APOE* ε4 carrier status, this observation was significant only among individuals carrying the *APOE* ε4 allele (Figure 2; Table 3).

No associations were observed for the cohort as a whole, or when stratified by *APOE* ɛ4 carrier status for interactions between MeDi and Western diet, and symptoms of depression or anxiety on brain Aβ load. Additionally, when stratified by *APOE* ε4 carrier status, there was no significant association between the DASH diet and symptoms of depression on brain Aβ load (Table 3).

## 4 DISCUSSION

This study investigated the moderating effect of dietary patterns on the relationship between symptoms of depression and anxiety, and brain Aβ load in a cohort of cognitively unimpaired older adults.

The DASH diet was observed to moderate the relationship between symptoms of depression and brain Aβ load, such that in those with lower DASH diet consumption, greater depressive symptoms were associated with higher Aβ load. Additionally, the DASH diet was observed to moderate the relationship between anxiety symptoms and brain Aβ load, such that in those with lower DASH diet consumption, greater anxiety symptoms were associated with higher Aβ load. This association was observed in the cohort as a whole and in *APOE* ε4 allele carriers when stratified by *APOE* ε4 carrier status. Interestingly, the MeDi and Western diet scores did not moderate the relationships between symptoms of depression and anxiety and brain Aβ load in the cohort as a whole or when stratified by *APOE* ε4 carrier status. Given that anxiety has been shown to interact with Aβ burden to accelerate cognitive decline, dietary interventions such as increasing DASH diet adherence may serve as a modifiable factor to mitigate this effect.^3^, ^10^, ^13^ These findings further support the evidence that dietary patterns, particularly the DASH diet, may moderate the association between symptoms of mental health (depression and anxiety) and AD brain biomarkers.

Several studies have provided evidence supporting the notion that lower adherence to the DASH diet is associated with increased depression and poorer mental health outcomes.^40^, ^41^ In previous studies, even individuals with low adherence to the DASH diet demonstrated a significant inverse association between DASH diet adherence and depressive symptoms and mental health outcomes.^40^, ^42^ This finding suggests that incorporating elements of the DASH diet into one's eating habits, rather than strict adherence, may still offer mental health benefits. Moreover, research has indicated that individuals dealing with mental illnesses often face challenges in maintaining a healthy diet, potentially leading to lower adherence to healthy diets like the DASH diet.^43^ This lowered adherence may exacerbate the negative impact of mental illnesses on cognitive health, potentially contributing to increased Aβ deposition.^44^, ^45^ Conversely, research indicated that the DASH diet had a beneficial influence on Aβ levels, potentially reducing the accumulation of Aβ related to AD.^46^

The significant interaction observed whereby adherence to the DASH diet at lower than the mean level was moderating the relationship between anxiety symptoms and Aβ burden specifically among *APOE* ɛ4 allele carriers, suggests that those at higher risk of AD due to carriage of the *APOE* ɛ4 allele, were also at greater risk for diet‐related Aβ deposition if they also had anxiety symptoms. Lower adherence to the DASH diet may exacerbate the negative effects of anxiety symptoms on cognitive health. This aligns with the findings of Smith et al.,^43^ which suggest that anxiety, through mechanisms such as unhealthy dietary choices and stress‐related neurobiological pathways, could potentially accelerate Aβ deposition and hasten the progression of AD in *APOE* ɛ4 allele carriers.

Importantly, we observed that neither the MeDi nor Western diet scores showed significant interactions with symptoms of depression, anxiety, or brain Aβ load. These results indicate that the DASH diet may exert a unique influence compared to other dietary patterns assessed in this study. One possible explanation for the lack of significant interactions with the MeDi diet score could be the diverse nature of this dietary pattern. Although the MeDi diet emphasizes heart‐healthy fats like olive oil, nuts, and fish, individuals following the diet may have varying nutritional compositions and dietary choices.^47^ This variability may have weakened the potential associations between the MeDi diet and symptoms of depression and anxiety, and brain Aβ load in our cohort. Similarly, the Western diet score, which reflects a dietary pattern characterized by high levels of processed foods, saturated fats, and sugar, did not exhibit significant moderating effects. This may be due to the broad spectrum of Western dietary habits, as individuals within this group might still have divergent nutrient intakes and dietary preferences that impact the relationship between diet and cognitive outcomes.^48^, ^49^ Contrary to this, the DASH diet specifically focuses on reducing sodium intake while promoting the consumption of nutrient‐rich foods like fruits, vegetables, whole grains, lean proteins, and low‐fat dairy products.^50^, ^51^ These components are known to have profound effects on blood pressure regulation and overall vascular health. It is possible that the precise and targeted nature of the DASH diet's recommendations, particularly its emphasis on specific nutrients such as potassium, calcium, and magnesium, could explain its unique impact on symptoms of depression and anxiety, and brain Aβ load observed.^52^, ^53^ A central feature of the DASH diet is its emphasis on reducing sodium intake. Excessive sodium consumption has been associated with endothelial dysfunction, increased blood pressure, and blood–brain barrier (BBB) disruption, which can all impair cerebral perfusion and promote Aβ accumulation in the brain.^54^, ^55^ Studies have shown that high sodium intake can exacerbate neurovascular dysfunction and may lead to impaired Aβ clearance across the BBB, thereby accelerating Aβ pathology.^56^ In contrast, adherence to the DASH diet, characterized by low sodium intake, may improve vascular health^57^ and facilitate more efficient clearance of Aβ from the brain.^53^ Furthermore, the DASH diet is rich in magnesium, a mineral that plays a crucial role in neuroprotection by regulating N‐methyl‐D‐aspartate (NMDA) receptor activity, an important mechanism given that excessive NMDA receptor activation can lead to excitotoxicity and neuronal damage, which are implicated in the pathogenesis of AD, while also modulating oxidative stress, and enhancing synaptic plasticity.^58^, ^59^ Magnesium has also been shown to inhibit Aβ aggregation and reduce neuroinflammation, two key pathological features of AD.^60^ Additionally, potassium and calcium, also abundant in the DASH diet, contribute to improved vascular function^61^ and reduced oxidative stress.^62^ These findings highlight the necessity for personalized dietary interventions, taking into account individual preferences and genetic factors, to enhance the precision and effectiveness of dietary strategies for cognitive health.^63^ The other main difference is alcohol; current research on the relationship between alcohol consumption and the risk of AD has not yet yielded a definitive conclusion. Studies suggest a potential link between high alcohol consumption and an increased risk of AD,^64^, ^65^, ^66^ with others observing a protective effect associated with moderate alcohol intake.^67^, ^68^ Although MeDi involves only low–moderate alcohol intake, this potentially could have a negative impact on our cohort.

The study has limitations to acknowledge. The CCVFFQ relies on participants’ estimations of food intake over the previous year; this can lead to misclassification of dietary pattern adherence due to limited accuracy and is a common limitation in studies with diet. To address this limitation, we assessed participants classified as cognitively unimpaired only. As part of the study exclusion criteria, those exhibiting significant current depression at the time of the baseline assessment (GDS score > 5) were excluded. Therefore, the cohort used in the current analysis had only a possibility of mild depression, and additional associations may have been observed in a cohort, including those with moderate and severe depression levels. The exclusion criteria for specific conditions and the focus on cognitively unimpaired individuals may limit the generalizability of findings to broader populations, including those with cognitive impairment. Additionally, although *APOE* ε4 carrier status was accounted for, an insufficient number were ε4 homozygotes (*n* = 16), limiting our ability to explore dose‐dependent *APOE* effects. Finally, the study's cross‐sectional design limits the inference of temporal and cause‐and‐effect relationships, and this will need to be assessed using longitudinal and intervention data. To strengthen causal inferences and clarify the directionality of observed associations, future research will use the longitudinal design of the AIBL study to examine whether adherence to specific dietary patterns and the presence of depressive or anxiety symptoms predict changes in cerebral Aβ burden over time. These analyses will provide critical insights into the temporal dynamics underlying the relationship between modifiable lifestyle factors, mental health, and AD pathology.

Many aspects of our study provide confidence in our findings. We have used a well‐characterized cohort with the vast majority being White, increasing the internal validity of our results. We have taken a very conservative approach by controlling for a wide range of demographic variables and using FDR adjustment to determine statistical significance. We also tested the inclusion of physical activity level as a potential confounder and found that it did not alter the results. The dietary data were collected using an instrument previously validated in earlier epidemiological studies to comprehensively evaluate long‐term dietary intake.^69^

Our study provides valuable insights into the complex relationships among dietary patterns, mental health symptoms (depression and anxiety), and an AD‐related brain biomarker as assessed through PET imaging. The findings highlight the moderating role of the DASH diet in this complex relationship in older adults. Low adherence to the DASH diet appears to exacerbate the negative impact of mental health symptoms on brain health, underscoring the importance of considering dietary interventions in the context of mental health and neurodegenerative disease prevention and management. Additionally, the interaction observed only in *APOE* ε4 allele carriers between anxiety symptoms and brain Aβ burden in those with a lower DASH diet score highlights the role of genetics in this relationship. Importantly, the unique effects of the DASH diet compared to other dietary patterns suggest the need for more tailored dietary interventions in future research. Overall, this study contributes to our understanding of the intricate interplay among diet, mental health symptoms, and neurodegenerative processes.

## CONFLICT OF INTEREST STATEMENT

The authors declare no conflicts of interest related to this work. The Australian Imaging, Biomarkers, and Lifestyle (AIBL) study has received partial financial support from various funding sources, including government and non‐government organizations, as outlined in the funding section. Some authors are affiliated with institutions that have received research funding from industry partners; however, none of these relationships influenced the study design, data collection, analysis, interpretation, or manuscript preparation. All authors have reviewed and approved the final manuscript and declare no competing interests. Author disclosures are available in the supporting information.

## CONSENT STATEMENT

This study was conducted in accordance with the ethical standards of the participating institutions and the Declaration of Helsinki. Ethical approval was obtained from the institutional ethics committees of Austin Health, St. Vincent's Health, Hollywood Private Hospital (now Ramsay Health Care), and Edith Cowan University. All participants provided written informed consent before undergoing any study‐related procedures.

## Supporting information



Supporting Information

## Data Availability

The data underlying this article were accessed from the Australian Imaging, Biomarkers, and Lifestyle (AIBL) study and are not publicly available due to ethical and privacy restrictions. Access to AIBL data can be requested through the AIBL study website (https://aibl.csiro.au) following their data access procedures. Additional information can be obtained by contacting the corresponding author.
